# Promising Role of Circulating Tumor Cells in the Management of SCLC

**DOI:** 10.3390/cancers13092029

**Published:** 2021-04-22

**Authors:** Antonella De Luca, Marianna Gallo, Claudia Esposito, Alessandro Morabito, Nicola Normanno

**Affiliations:** 1 Cell Biology and Biotherapy Unit, Istituto Nazionale Tumori—IRCCS—Fondazione G. Pascale, 80131 Naples, Italy; a.deluca@istitutotumori.na.it (A.D.L.); marianna.gallo@istitutotumori.na.it (M.G.); claudia.esposito@istitutotumori.na.it (C.E.); 2 Thoracic Medical Oncology Unit, Istituto Nazionale Tumori—IRCCS—Fondazione G. Pascale, 80131 Naples, Italy; a.morabito@istitutotumori.na.it

**Keywords:** small-cell lung cancer, circulating tumor cells, chemotherapy, prognostic biomarker, targeted agents

## Abstract

**Simple Summary:**

Despite the recent approval of immune-checkpoint inhibitors, therapeutic strategies for the treatment of small-cell lung cancer (SCLC) patients remained unchanged for decades. The aggressiveness of the disease and the lack of active treatments underlie the need for the identification of biomarkers that can drive therapeutic decisions. Here we discuss the potential role of circulating tumor cells in SCLC research as a promising tool for improving the clinical management of SCLC patients.

**Abstract:**

Small cell lung cancer is an aggressive disease for which few therapeutic options are currently available. Although patients initially respond to therapy, they rapidly relapse. Up to today, no biomarkers for guiding treatment of SCLC patients have been identified. SCLC patients rarely undergo surgery and often the available tissue samples are inadequate for biomarker analysis. Circulating tumor cells (CTCs) are rare cells in the peripheral blood that might be used as surrogates of tissue samples. Different methodological approaches have been developed for studies of CTCs in SCLC. In addition to CTC count, which might provide prognostic and predictive information, genomic and transcriptomic analyses allow the characterization of molecular profiles of CTCs and permit the study of tumor heterogeneity. The employment of CTC-derived xenografts offers complementary information to genomic analyses and CTC enumeration about the mechanisms involved in the sensitivity/resistance to treatments. Using these approaches, CTC analysis is providing relevant information on SCLC biology that might aid in the development of personalized therapeutic strategies for SCLC patients.

## 1. Introduction

Small cell lung cancer (SCLC) is the most aggressive lung cancer subtype and represents about 13% of all new diagnosed lung cancers [1]. SCLC is a disease characterized by neuroendocrine features, a rapid tumor cell growth and the tendency to disseminate early. The majority of patients (about 70%) presents an extensive stage disease (ES-SCLC) at diagnosis, the remaining 30%, a limited stage of disease (LS-SCLC). The prognosis of SCLC is poor, with a median overall survival (OS) of 10 months for patients with ES-SCLC and a survival up to 4 years for selected patients with LS-SCLC [2].

Platinum-based chemotherapy in combination with etoposide or irinotecan is the standard first-line treatment. Recently, immune-checkpoint inhibitors (ICIs) alone or in combination with chemotherapy have been approved for the treatment of SCLC [3].

Despite most patients initially responding to chemotherapy, alone or in combination with ICIs, with a high response rate, a rapid recurrence frequently occurs with an unfavorable prognosis [4]. The only approved second-line agent topotecan is associated with a low response rate and a short duration of survival [2]. Unlike non-small cell lung cancer (NSCLC) and other cancer types, in SCLC there are few therapeutic options and no targeted therapies are available for the management of patients in an advanced stage of disease.

Genomic profiling of SCLC revealed a high load of somatic mutations (about 8 mut/Mb) and molecular signatures associated with tobacco smoking, which plays a pivotal role in the pathogenesis of the disease [5,6]. Biallelic inactivation of *TP53* and *RB1* are nearly ubiquitary in SCLC [6]. Mutations in other genes, including *CREBBP*, *EP300*, *NOTCH1*, and amplification of *MYC* and *SOX* family genes, *FGFR1* and *IRS2* have been also observed [6,7]. Fusion genes, including a recurrent *RLF1-MYCL1* fusion, have been also reported [7]. Recently, a molecular classification, based on gene expression profiling, of four distinct SCLC subtypes characterized by the differential expression of four transcription factors, achaete-scute homologue 1 (*ASCL1*), neurogenic differentiation factor 1 (*NeuroD1*), yes-associated protein 1 (*YAP1*) and POU class 2 homeobox 3 (*POU2F3*) has also been proposed [8]. Despite the genomic complexity of SCLC, few actionable mutations that offer potential for therapeutic intervention with targeted therapy have been identified in SCLC patients.

The high aggressiveness of this disease and the lack of active treatments underlie the need for the identification of biomarkers that can aid in the development of personalized medicine in SCLC. In this respect, SCLC patients rarely undergo surgery and tissue samples obtained for diagnosis are often inadequate for biomarker analyses. Non-invasive biomarkers in peripheral blood, including circulating tumor cells (CTCs) or cell free DNA (cfDNA), can offer the opportunity to achieve prognostic and/or predictive information, to study mechanisms of resistance and to discover novel targets for therapeutic approaches. Although cfDNA testing is the most advanced approach in clinical routine, a great number of the studies are focused on CTCs in SCLC [9].

CTCs are rare cells released from primary tumors and/or metastatic sites into peripheral blood (one CTC per 10^6^–10^7^ white blood cells) with a short half-life [10]. Patients with SCLC have a relatively higher CTC number as compared to NSCLC patients [11] and patients with ES-SCLC have more CTCs compared to patients with limited disease [12,13,14,15].

In the last few years, technical advancements in isolation methods along with the possibility to recover and molecularly characterize single CTCs, have helped to assess the potential role of CTCs as biomarker for monitoring disease progression in order to study tumor heterogeneity and understanding of the mechanisms of resistance to therapies. In addition, the employment of CTC-derived xenograft (CDX) models has allowed performing studies into SCLC biology in vivo.

In this review, we will discuss the different methodological approaches employed in CTC studies and their utility in improving the management of SCLC patients.

## 2. Methodological Approaches to CTC Studies in SCLC

Several technologies have been developed for CTC enrichment and detection (Table 1). The most widely used platform for CTC analysis is the CellSearch System, which allows CTC isolation and enumeration based on their expression of EpCAM, a cell surface marker overexpressed in many epithelial tumors [16]. Although SCLC often displays a neuroendocrine differentiation, the expression of EpCAM has been described in SCLC cells [17,18]. In this respect, our group was the first to demonstrate that the CellSearch System is able to isolate EpCAM-positive CTCs in SCLC patients [19]. Our original finding has been later confirmed by a number of studies [14,15,20,21]. However, other approaches have been developed to improve the capture of CTCs with low or without expression of epithelial markers, which might result in a higher efficiency in isolating CTCs from SCLC patients (Table 1).

These methods have been described in several review articles [16,22,38]. Some approaches, based on the expression of cell surface markers, allow the positive or negative enrichment of CTCs in SCLC samples, including the possibility to recover viable CTCs [23,24] (Table 1).

Methods based on physical properties, such as size and deformability, have the advantage of enriching CTCs with epithelial and mesenchymal features [26,28,32,33]. Other technologies, such as the CTC-iChip, combine physical and biological properties for the enrichment of both epithelial and non-epithelial CTCs [30]. Some platforms, such as the DEPArray, can isolate single CTCs after enrichment with other methods [35]. Among alternative approaches to isolate CTCs from SCLC, the TelomeScan assay employs a green fluorescent protein (GFP) gene-expressing adenovirus in which telomerase regulates viral replication. As telomerase activity is higher in cancer cells rather than in normal cells, GFP-positive CTCs can be efficiently isolated [36,37] (Table 1).

After the enrichment step, CTCs can be detected and characterized using immunologic, molecular and functional assays.

**Figure 1:**
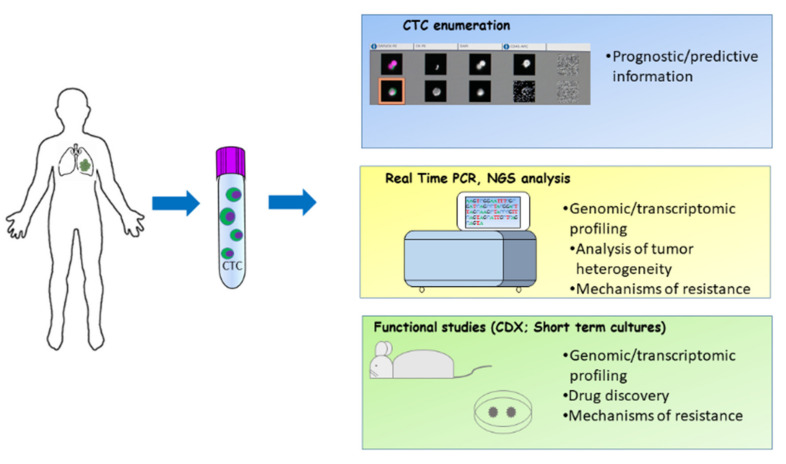
Methodological approaches to study CTCs in SCLC. CTCs enriched and isolated with various techniques offer the opportunity to perform different downstream assays such as CTC count, molecular analyses and in vivo functional studies.

Isolated CTCs offer different opportunities for studies in SCLC. In addition to CTC count that may provide prognostic and predictive information, molecular profiling of CTCs might allow the identification of biomarkers of sensitivity/resistance to therapy and deliver information on tumor heterogeneity. In addition, preclinical studies using CDXs and ex-vivo CTCs may offer the opportunity to acquire information on SCLC biology and facilitate the discovery of novel therapeutic approaches (Figure 1).

### 2.1. CTC Count as Biomarker in SCLC

A number of studies have addressed the prognostic role of CTC count in patients with SCLC (Table 2). It is very difficult to summarize the main findings of these studies because of their high heterogeneity.

Although the CellSearch System has been the most used platform in studies assessing CTC count as a prognostic biomarker in SCLC, other technologies such as TelomeScan and methods based on negative immunomagnetic enrichment and immunocytochemistry have been also used [39,40] (Table 2). These approaches are based on different technologies and might detect different populations of CTCs, making their comparison difficult. Taking into account these considerations, we will focus our discussion only on studies that employed the CellSearch system, which still has several limits.

While three studies with the CellSearch enrolled only ES- and one only LS-SCLCpatients, the majority of the studies enrolled both ES- and LS-SCLC patients (Table 2). Patients with LS disease have a better prognosis as compared with patients with ES disease [2,46]. In addition, patients with ES disease have a number of CTCs, significantly higher than patients with LS-SCLC, thus making extremely heterogeneous the population of patients in studies that included both ES- and LS-SCLC [13,14,15,21]. The importance of the heterogeneity of the population of patients is confirmed by some studies that reported a prognostic value of CTC count only in the subgroup of patients with ES disease [14,21]. Only one study found that the CTC number at baseline is an independent prognostic factor for PFS and OS in LS patients treated with chemoradiotherapy [45].

The number of patients enrolled is limited in most studies, ranging between 14 and 120 (Table 2). The time points of CTC assessment are also different among the studies. In particular, in addition to the CTC count performed before the treatment, CTCs were collected at various days after treatment, after a various number of treatment cycles, and/or at progression. Finally, patients enrolled in the studies were subjected to different therapeutic regimens, i.e., chemotherapy or chemoradiotherapy.

Most studies employed one or more cut-off values to discriminate between patients with a high versus low CTC count. However, such cut-off values varied significantly. The identification of the optimal cut-off was influenced by the statistical methods employed for calculation, the size of samples, the diverse treatment regimens and, most likely, the fraction of ES vs. LS patients enrolled. All these variables might indeed explain the different cut-off values used to discriminate patients with a poor versus a good prognosis [20,41].

Although the above-described heterogeneity significantly limits the possibility to compare the results of the different studies on the prognostic role of CTC count in SCLC, some general findings are common to most of the reports published up to now.

All studies demonstrated that CTCs are detectable in most SCLC patients at baseline (i.e., before treatment), and that the number of CTCs is usually higher in SCLC as compared with most solid tumors [12] (Table 2).

More importantly, the majority of the studies are concordant in identifying a high baseline CTC count as a relevant prognostic factor in SCLC patients. Indeed, the CTC number at baseline was confirmed to be an independent prognostic factor for PFS and OS at multivariate analysis [15,21,41,45]. This evidence was also supported in a meta-analysis of seven studies enrolling 440 SCLC patients, in which a strong association between the presence of CTCs at baseline and a poor clinical outcome was demonstrated [47].

Although the timing for CTC enumeration after the treatment varied among the studies, the majority of the trials also found that the CTC count after one or more cycles of treatment predicts the outcome of SCLC patients. In the study of Hou and collaborators, a number of CTCs < 50 after one cycle of chemotherapy was associated with longer PFS and OS [41]. In a different study, patients with a CTC count ≥8 after treatment and at relapse had a worse OS as compared with those having <8 CTCs at the same time points [14]. Furthermore, the analysis of CTCs in 59 patients before, after one cycle and at the end of chemotherapy revealed that a number of CTCs < 2, after the first or the fourth cycle of chemotherapy, was a strong predictor for PFS and OS, although at multivariate analysis only the absolute number after the first cycle remained the most significant marker for OS [15]. Other studies showed that the CTC number after the second cycle of treatment is also a strong predictor of the outcome [21,42]. In the study of Messaritakisand collaborators, only the detection of CTCs at progression was considered an independent prognostic factor for OS at multivariate analysis [20].

Importantly, the change in the CTC number after chemotherapy was found to be a strong predictor of survival in different studies [14,41,43]. In particular, a study from our group in 60 ES SCLC patients suggested that the accuracy of the prognostic model was only marginally increased by the addition of CTC count to clinical information, whereas a reduction of CTCs greater than 89% following the first cycle of therapy had the strongest correlation with a lower risk of death (HR 0.24) with a significant increase of the prognostic accuracy [43]. These findings strongly suggest that CTC reduction might reflect the chemosensitivity of SCLC.

Although a correlation between CTC number and outcome was clearly demonstrated, a relationship between CTC count and response to treatment in SCLC patients was not found. In the study by Hiltermann, the decrease in CTC number from baseline to after one cycle of chemotherapy did not correlate with tumor response [15]. Similarly, Naito and colleagues did not find a significant correlation between response to treatment and the CTC number before and after chemotherapy [14]. These results are in agreement with the study of Aggarwal and colleagues who did not found a significant correlation between decrease in CTCs and a response to chemotherapy [21].

Clinical studies evaluating novel therapeutic agents for SCLC patients have planned CTC analysis as prognostic/predictive biomarker (Table 3). These studies employed the CellSearch system for CTC isolation and enumeration.

In particular, in a clinical trial of the multi-kinase inhibitor pazopanib in patients with recurrent/refractory SCLC, a number of CTCs ≥ 5 was detected in 28/56 (50.0%) of patients [48]. Treatment with pazopanib for one cycle significantly decreased the number of patients with a high CTC number. Patients with PD as the best response had a significantly higher number of CTCs at baseline as compared with patients experiencing PR or SD. At multivariate analysis, an increased number of CTCs after one cycle was associated with poor OS [48].

An exploratory analysis of the predictive role of CTCs was performed in a phase II clinical trial enrolling 78 ES-SCLC patients who received chemotherapy plus the CXCR4 antagonist LY2510924 [49]. A CTC number ≥6 and a percentage of CXCR4-positive CTCs ≥ 7% were considered optimal cut-off values, based on receiver operating characteristic (ROC) analysis. A CTC number ≥6 at baseline and at cycle 2 predicted shorter PFS and OS. A frequency of CXCR4-positive CTCs > 7% at baseline was also a prognostic factor for shorter PFS [49].

The predictive role of CTCs was also explored in a randomized phase II study evaluating the efficacy of the Hedgehog inhibitor vismodegib or the insulin-like growth factor 1 receptor antibody cixutumumab in combination with standard chemotherapy in previously untreated patients with ES-SCLC [50]. Patients with a CTC number >100 at baseline (39/120, 32.5%) had a worse OS as compared with patients with a lower CTC count [50].

Finally, in a phase I clinical trial investigating the combination of the Hedgehog inhibitor sonidegib with standard chemotherapy in untreated ES-SCLC patients, CTCs were isolated and enumerated with the CellSearch System before, during and at disease progression [51]. Elevated CTC count at baseline (>200) was associated with worse OS at univariate analysis. A persistently high CTC number at cycle 2 also correlated with worse OS. An increase in CTCs from the nadir to progression was observed in 5/13 patients [51].

### 2.2. Molecular Characterization of CTCs in SCLC

Real time (RT)-PCR techniques were used for the detection of specific markers in CTCs isolated from SCLC patients. In this regard, the presence of transcripts of epithelial (*EpCAM* and *CK19*) and neuroendocrine (*CHGA, SYP, NCAM1* and enolase 2, *ENO2*) markers in CTCs enriched with a microfluidic system was investigated in a study enrolling 48 SCLC patients [52]. The expression of the neuroendocrine markers *SYP* and/or *CHGA* at diagnosis and at disease progression correlated with worse OS [52]. However, these results should be confirmed in additional studies. Interestingly, RT-PCR also revealed in 7.8% SCLC patients the presence of the delta-like 3 ligand (*DLL3*) transcript belonging to the Notch pathway and associated with neuroendocrine tumorigenesis. DLL3-positive patients had a significantly shorter OS than DLL3-negative patients (median OS 2 vs. 7 months) [52].

The employment of Next-Generation Sequencing (NGS) approaches that can interrogate a large number of genes in a single analysis, along with the development of technologies that allow isolating single CTCs, such as the DEPArray system, offered the possibility to perform a comprehensive genomic/transcriptomic profile of CTCs isolated from SCLC patients. Whole genome sequencing (WGS) of single CTCs enriched with the CellSearch System and individually isolated under a fluorescence microscope revealed that copy number variation (CNVs) profiles are specific for each cancer type [53]. In particular, the CNV profile of CTCs reflects the genetic landscape of metastasis and is highly reproducible from cell to cell and from patient to patient, in contrast with whole exome sequencing (WES) analysis of single nucleotide variations (SNV) and insertions/deletions (indels) that are highly heterogeneous from cell to cell [53].

The molecular profile of single CTCs from 13 SCLC patients, enriched with the CellSearch system and isolated using the DEPArray technology, was analyzed by WGS to generate 16 copy number alteration (CNA) profiles that stratified patients in chemosensitive or chemorefractory [54]. The CNA classifier was subsequently validated in an additional 18 patients. The CTC CNA classifier correctly assigned 83.3% of the cases as chemorefractory or chemosensitive. A homogeneous CNA classification was observed in the majority of patients (19/31). However, in 12/31 cases, intra-patient heterogeneity among single isolated CTCs was observed. When the CTC CNA classifier was applied before treatment, a statistically significant difference in PFS of chemosensitive compared to chemorefractory patients (median PFS, 2.8 months for chemorefractory; 5.8 months for chemosensitive; *p* value = 0.0166) was observed, suggesting a potential clinical utility of the CNA classifier. However, no changes were observed in CNA profiles in CTCs isolated at baseline from patients initially chemosensitive and CTCs isolated upon progressive disease, suggesting that other mechanisms may regulate the acquired resistance to chemotherapy [54].

In another study, single CTCs from 48 SCLC patients captured with the CellSearch were subjected to WES analysis to identify SNVs and indels and to WGS for CNA profile detection [55]. Ten CNA regions were selected for the establishment of a CNA score from CTCs obtained before treatment, as classifier for predicting the outcome of SCLC patients. Patients with a low CNA score (<0) after the first-line chemotherapy had a longer PFS and OS as compared with patients with a higher score (≥0). Multivariate analysis showed that a high CNA score was an independent predictor of poor PFS and OS. Interestingly, the authors found an increase in genomic heterogeneity during disease progression, due to the allelic loss of CNAs in CTCs [55].

### 2.3. Functional Studies of CTCs in Preclinical Models

Functional analyses using preclinical models may offer complementary information to both genomic analyses and CTC count about the biology of SCLC and the discovery of therapeutic targets. The main requirement for these experiments is the isolation of viable CTCs. Functional studies of CTCs in mouse models are mainly performed using two approaches: the direct injection of CTCs into mice to generate CDX models or the establishment of cultures of CTCs ex-vivo.

Hodgkinson and colleagues was the first to demonstrate that CTCs isolated from SCLC patients are tumorigenic when injected in immunocompromised mice [56]. NGS analysis of CDXs confirmed a genomic profile characteristic of SCLC and showed a patient-specific pattern of CNA gains and losses, with the loss of *RB1*, *TP53* and *PTEN*, commonly observed in SCLC. Moreover, the response of CDXs to cisplatin and etoposide was closely correlated with the outcome of the corresponding patients. The comparison of the genomic profiles of single CTCs with the corresponding CDX indicated a high correlation between CDXs and CTCs, despite in one patient heterogeneous CNA profiles between single CTCs being observed [56].

An automated microfluidic apparatus for viable CTC isolation was employed to generate CDXs with an efficiency of tumor growth in nude mice of 38% and a median latency of 112 days [57]. CTC-derived models retained a stable genome and the same alterations during serial passages, demonstrating to recapitulate the donors’ tumors. Etoposide sensitivity in these models correlated with the clinical behavior of SCLC patients. Transcriptomic analysis revealed a *MYC* signature that was strongly correlated with etoposide resistance [57].

CDX models from SCLC patients with different sensitivity to chemotherapy have been used to analyze the mechanisms of resistance [58]. RNA-Seq analysis of CDX-derived single cells revealed the presence of neuroendocrine markers (*ASCL1, NEUROD1*), of *MYC* family genes and elevated epithelial-to-mesenchymal transition (EMT) scores. A high intratumor heterogeneity was described in chemotherapy-resistant CDXs at baseline, with upregulation of multiple signaling pathways associated with platinum resistance (including MYC, WNT and EMT pathways) within the same tumor. CTCs and CDXs collected at relapse were demonstrated to be more heterogeneous than at the time of diagnosis, suggesting that intratumor heterogeneity might be involved in the resistance to therapy [58].

CDXs from CTCs have the advantages of generating a large number of xenografts from patients for which tissue samples are not available and are able monitor the course of disease in a non-invasive manner. However, this approach has some limitations, such as the long time occurring to generate mouse models, the high cost of the in vivo pharmacology experiments and ethical implications. Ex vivo cultures of CTCs allow the generation of models in a shorter period with reduced costs. Ex vivo cell lines have been established from CTCs isolated in different cancer types, including breast, colon cancer and SCLC [59,60,61]. CTCs isolated from patients with extended SCLC allowed generation of ex-vivo cultures characterized by the presence of spheroidal morphology and stem cell markers that form tumorospheres with a mesenchymal-epithelial transition (MET) phenotype under culture [62]. Ex vivo cell lines resulted in being more sensitive to epirubicin and showed elevated cytotoxicity in response to the combination of epirubicin and topotecan as compared to SCLC continuous cell lines [63]. When CTC-derived cell lines spontaneously developed tumorospheres, the sensitivity to epirubicin and topotecan was reduced [64].

Finally, a recent study used CDX-derived cells to develop ex vivo short-term cultures [65]. CDX-derived cell lines maintained the same phenotypic and molecular characteristics of the corresponding CDXs. The response of ex vivo cell lines to chemotherapy correlated with the response observed in in vivo experiments. In addition, the authors demonstrated that short-term cultures generated from CDXs are a suitable approach for testing novel targeted agents [65].

## 3. Open Questions and Future Perspectives

SCLC is a highly aggressive subtype of lung cancer and its management is challenging, due to the rapid course of the disease and to the limited therapeutic options. The lack of tissue samples for preclinical and clinical studies has represented one of the major obstacles for studies about SCLC biology and drug development. Although the potential clinical utility of CTCs as surrogate of tumor tissue for prognostic and predictive information, for monitoring the course of the disease, and studying mechanisms of resistance has been demonstrated in different studies, CTC analysis is not currently employed in the clinical management of SCLC.

Several studies have demonstrated a prognostic role of the number of CTCs and/or the reduction of the absolute number of CTCs from baseline to first or subsequent cycles of chemotherapy [14,15,21,41,43,45]. However, the different technologies used for CTC enumeration, the heterogeneous patient populations included in the studies, a lack of a validated unique cut-off and the variability observed in the reduction of CTCs during the course of treatment, limited the utility of this biomarker in clinical practice. In this regard, the identification of a unique cut-off is a key issue for the development of CTC number as biomarker in SCLC. Indeed, not only have different techniques been employed for CTC analysis but also in the studies using the same technology (i.e., the CellSearch System) different cut-off values have been identified. Several factors may have influenced the identification of the threshold: (i) the low number of patients included in the majority of studies; (ii) the heterogeneity of the series analyzed with particular regard to the stage of the enrolled patients, given that patients with ES-SCLC generally have higher CTC levels than those of LS-SCLC patients; (iii) the different statistical approach used to identify the cut-off, often not justified by a priori hypotheses; (iv) the timing of the sampling which, with the exception of the baseline, was often performed at different times after the therapy. In addition, the majority of the studies in SCLC employed the CellSearch for CTC enrichment and isolation. However, the CellSearch technology is based on EpCAM for enrichment of CTCs and it might miss cells that have undergone an EMT phenotype. The employment of EpCAM-independent technologies might increase the detection rate of CTCs in SCLC. Nevertheless, the CTC count has been included in exploratory analyses in clinical studies evaluating novel targeted agents in SCLC [48,49,50,51], confirming the importance of the evaluation of the CTC number as a prognostic biomarker in this disease.

Molecular profiling of single CTCs confirmed the molecular complexity of SCLC characterized by the high tumor mutational burden, the ubiquitary presence of mutations in the *TP53* and *RB1* genes and a high number of CNAs [53,54,55,66]. Although it has been demonstrated that the CNA profiles of individual CTCs in each patient is homogeneous, some studies evidenced a heterogeneity at a single cell level both before and during the treatment, which might be associated with chemotherapy resistance [54,55]. However, to assess the involvement of intratumor heterogeneity in the evolution of the disease and the response to treatments, the genomic profile of a high number of single CTCs from multiple regions of the tumor or from different tumor sites at different time points should be analyzed. In this regard, the generation of CDXs and ex vivo cultures from CTCs might be of relevant importance in recapitulate tumor heterogeneity [67]. Interestingly, a study suggested that CDXs are more successfully generated from patients with a higher disease burden and a more aggressive disease [68]. Recently, transcriptomic analysis of a biobank of 38 CDXs was performed to analyze the mechanisms involved in tumor heterogeneity, confirming the presence of different molecular subtypes of SCLC [69].

**Figure 2:**
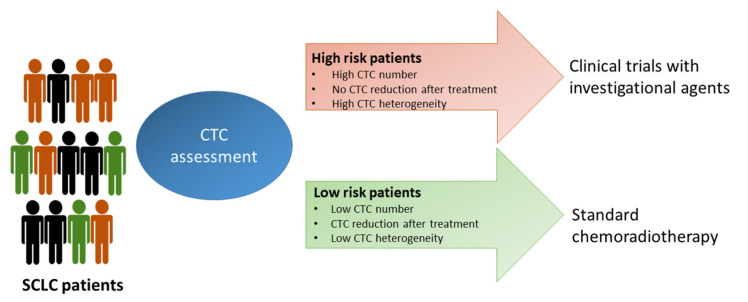
Possible SCLC patients’ stratification based on CTC analysis. High risk patients, based on their CTC status, could be enrolled in clinical studies with investigational drugs, whereas low risk patients could receive standard treatments.

The possibility to perform a molecular characterization of CTCs in combination with CTC count might provide information useful for patient selection in clinical studies. In this regard, patients with a high CTC number or a marginal reduction in the CTC number after the treatment and with a high level of intratumor heterogeneity could be enrolled in clinical trials with experimental agents, whereas patients with a low number of CTCs and with a homogeneous CTC population might be subjected to standard treatment (Figure 2).

A great potential of CTCs is the development of preclinical models for testing novel compounds. Unlike NSCLC, no targeted therapies have been developed in SCLC, due to the lack of actionable alterations in driver genes responsible of tumor development and progression. Different putative therapeutic targets are currently under investigation in SCLC, including DLL3, insulin-like growth factor 1 receptor (IGF1R), poly(ADP-ribose) polymerase (PARP), the DNA damage response (DDR) kinases ATM (ataxia-telangiectasia mutated), ATR (ATM- and Rad3-Related) and the cell cycle checkpoint kinases CHK1, WEE1 and aurora kinase A (AURKA) [70]. A number of compounds directed against these targets are in clinical development. Although preliminary data from clinical trials with agents targeting DLL3 showed modest clinical activity in heavily pre-treated SCLC patients [71], studies with novel agents and in the earlier phase of the disease will clear the relevance of DLL3 as a therapeutic target.

An association between the subtypes defined by the differential expression of ASCL1, NeuroD1, YAP1 and POU2F3 and specific targets have been identified [8], suggesting that specific subgroups of patients might benefit from these compounds. Interestingly, a recent study described in a CTC-derived mouse model a subtype switching that may be responsible for acquired resistance to chemotherapy [72].

## 4. Conclusions

A growing interest has recently emerged in the field of CTC research in SCLC for the potential utility of this biomarker in the clinic. CTC count coupled with genomic profiling might help to stratify patients for the optimal treatment. In addition, the analysis of the molecular profile of CTCs and the generation of CDXs are encouraging deeper knowledge of SCLC biology, with the major finding that SCLC is a very heterogeneous disease. The identification of different molecular subtypes and their vulnerability to unique pharmacological agents might aid in stratifying patients in clinical studies with investigational agents, with the aim to tailor a personalized treatment for each patient.

## Figures and Tables

**Table 1:** Overview of the main technologies used for enrichment and detection of CTCs in SCLC.

Technology [Refs]	CTC Enrichment	CTC Detection and Characterization	% of CTC Detection ^§^	Comments
**Protein marker-based devices**				
CellSearch System [12,22]	EpCAM antibodies-coated ferromagnetic beads	IF for CK8, 18, 19, DAPI and CD45	≥85%	FDA-approved semi-automated system. Do not detect EpCAM-negative CTCs. Do not recover viable cells.
CellCollector [23]	Functionalized medical wire associated with EpCAM antibodies	IF for EpCAM, CK and DAPI	Not applicable	CE-approved as medical device for in vivo CTC isolation. Capacity to process large volumes of blood with a high CTC detection rate.
RosetteSep System [24,25]	Depletion of leukocytes and erythrocytes by specific antibodies followed by density gradient centrifugation	ICC	46.9%	Fast and easy-to-use. Collection of live cells with high purity for many applications (cell cultures, DNA/RNA extraction, implantation in mice).
**Physical properties-based devices**				
ISET [26,27]	Size-based filtration for isolation of CTCs	IF; FISH	95%	Isolation of clusters and viable cells of epithelial and non-epithelial origin. Low recovery and purity.
ClearCell FX [28,29]	Microfluidic technology for CTC enrichment based on drag and size-dependent lift forces	IF; FISH	85%	Capacity to capture viable and intact CTCs for in vivo and in vitro experiments and for NGS analysis. Small CTCs may escape detection.
CTC-iChip [30,31]	Microfluidic platform for size-based isolation in combination with EpCAM-based positive selection or CD45 negative depletion	IF; RT-PCR for tumor associated transcripts	>77%	Detection of both epithelial and non-epithelial CTCs. Capture and in vitro culture of viable CTCs for functional studies.
Parsortix [32]	Microfluidic platform for cell size and deformability-based separation	IF for CK, DAPI and CD45	78%	CE-marked for use as in vitro diagnostic device. Collection of viable CTCs for molecular and functional analysis.
VTX-1 Liquid Biopsy System [33,34]	Microfluidic separation of CTCs based on cell size and deformability	IF; FISH, RT-PCR; NGS for tumor-associated transcripts	69%-79.5%	High recovery and purity of intact CTCs. No red blood cell lysis required. Suitable for many applications (genomic and proteomic analyses, enumeration, IF staining).
DEPArray [35]	Requires a pre-enrichment step with other technologies (e.g., CellSearch or Parsortix)	IF for CK, CD45, DAPI or Hoechst staining	99.7%	Recovery of single viable cells.
**Other Assays**				
TelomeScan [36,37]	Detection of GFP-positive CTCs following incubation with a telomerase-specific conditionally replicating adenovirus expressing the GFP gene	IF	>70%	Isolation of live CTCs, including EpCAM negative cells and cells undergoing EMT. A modified assay has been developed to reduce false-positive results, based on targeting miR-142-3p to inhibit GFP-expressing blood cells.

^§^ calculated by spiking tumor cells into peripheral blood of healthy donors. Abbreviations: EpCAM: epithelial cell adhesion molecule; IF: immunofluorescence; CK: cytokeratins; DAPI: 4’,6-diamidino-2-phenylindole; ICC: immunocytochemistry; FDA: US Food and Drug Administration; CTCs: circulating tumor cells; NGS: next-generation sequencing; RT-PCR: reverse transcriptase polymerase chain reaction; FISH: fluorescence in situ hybridization; DEP: Dielectrophoresis; GFP: green fluorescent protein.

**Table 2:** Selected studies assessing the role of CTC number as prognostic or predictive biomarker in SCLC.

Study [Ref]	Disease Stage	Treatment	Blood Sample Collection	Number of Patients	CTC Detection Method	Optimal Cut-Off	Main Findings
Hou et al. [13]	LS- and ES-SCLC	Chemotherapy	Baseline, days 2 and 22 after the treatment	50	CellSearch	No cut-off	Patients with a high number of CTCs (> 300) had a shorter median OS than patients with a low number of CTCs (< 2) (134 vs. 443 days). A persistently elevated CTC number at day 22 after treatment was considered an adverse prognostic factor at univariate analysis.
Hou et al. [41]	LS- and ES-SCLC	Chemotherapy	Baseline, post cycle 1	97	CellSearch	50 CTCs/7.5 mL blood	Patients with a CTC number > 50 had a shorter median PFS (4.6 versus 8.8 months) and OS compared to those with a CTC number < 50 (5.4 versus 11.5 months) at baseline. A number of CTC < 50 after one cycle of chemotherapy was associated with longer PFS and OS. At multivariate analysis, the CTC number at baseline was an independent prognostic factor for PFS (HR = 2.01) and OS (HR = 2.45).
Naito et al. [14]	LS- and ES-SCLC	Chemotherapy or chemoradiotherapy	Baseline, post treatment, at relapse	51	CellSearch	8 CTCs/7.5 mL blood	Patients with a CTC count < 8 at baseline had longer OS than patients with CTC ≥8. Patients with a CTC count ≥8 after treatment and at relapse had a worse OS as compared with those with <8 CTCs at the same time points.
Hiltermann et al. [15]	LS- and ES-SCLC	Chemotherapy	Baseline, post cycle 1 and 4	59	CellSearch	2 CTCs/7.5 mL blood	Patients with a CTC count < 2 had longer OS than patients with a CTC number > 215 (729 vs. 157 days). At multivariate analysis, CTC count was an independent prognostic factor for PFS and OS at all time points. No correlations were observed between the decrease in CTC number from baseline to after one cycle of chemotherapy, and/or the absolute number of CTCs after one cycle of chemotherapy and response to treatment.
Cheng et al. [42]	ES-SCLC	Chemotherapy	Baseline, post cycle 2 and at progression	91	CellSearch	10 CTCs/7.5 mL blood	Patients with a CTC count ≥ 10 at baseline had significantly shorter OS as compared with patients with a CTC count < 10 (8.2 vs. 16.6 months); no difference in PFS between the groups was observed.
Aggarwal et al. [21]	LS- and ES-SCLC	Chemotherapy or chemoradiotherapy	Baseline, during cycles 1, 2 (days 2, 3), 3,4 (day 1) and at relapse	50	CellSearch	5 CTCs/7.5 mL blood 50 CTCs/7.5 mL blood	Patients with a CTC count < 5 at baseline had better PFS than patients with CTCs ≥ 5 (11 vs. 6.7 months). Using a cut-off of 50 CTCs, for patients with <50 CTCs, PFS and OS were both significantly longer compared to patients with CTCs ≥ 50. At multivariate analysis, a higher CTC count at baseline was associated with a high hazard of death and progression. The decrease in CTCs during the course of therapy was not significantly associated with the response.
Messaritakis et al. [20]	LS- and ES-SCLC	Chemotherapy	Baseline, after 1 cycle and at progression	83	CellSearch	5 CTCs/7.5 mL blood	Patients with a high number of CTCs had a significantly shorter median PFS and OS compared to patients with a low number of CTCs, irrespective of the time of CTC enumeration. At multivariate analysis, the detection of CTCs at baseline was considered as an independent factor associated with decreased PFS, whereas CTC count at progression was associated with a reduced OS. A significantly higher number of CTCs at baseline was observed in patients with PD compared to patients who experienced a CR/PR or SD.
Normanno et al. [43]	ES-SCLC	Chemotherapy	Baseline, post cycle 1	60	CellSearch	No cut-off	A CTC count reduction higher than 89% following chemotherapy was associated with a lower risk of death.
Huang et al. [44]	ES-SCLC	Chemotherapy	Baseline and within 4 weeks after chemotherapy	26	CellSearch	No cut-off	A trend toward significance was observed between baseline CTCs and the percentage of change from post-treatment to baseline and OS
Igawa et al. [39]	LS- and ES-SCLC	Chemotherapy or chemoradiotherapy	Baseline, at cycle 2 and 3, post cycle 4 and at progression	30	TelomeScan	2 CTCs/7.5 mL blood	Patients with a baseline CTC count < 2 had a significantly longer OS than patients with a CTC count ≥ 2.
Wang et al. [40]	LS- and ES-SCLC	Chemotherapy	Baseline, post cycle 1	42	Negative immunomagnetic enrichment	2 CTCs/7.5 mL blood	A CTC number ≥2 at baseline and after the first cycle of chemotherapy was significantly associated with worse PFS.
Tay et al. [45]	LS-SCLC	Chemoradiotherapy	Baseline	75	CellSearch	2 CTCs/7.5 mL blood 15 CTCs/7.5 mL blood 50 CTCs/7.5 mL blood	A number of 2 or 15 or 50 CTCs at baseline significantly correlated with PFS and OS. Patients with a CTC number < 15 had a better median PFS (19.0 months vs. 5.5 months) and OS (26.7 months vs. 5.9 months) than patients with a CTC number ≥15. At multivariate analysis only the 15 CTC cut-off emerged as an independent prognostic marker

Abbreviations: limited stage disease (LS-SCLC); extensive stage disease (ES-SCLC); progression-free survival (PFS); overall survival (OS).

**Table 3:** Clinical studies incorporating exploratory CTC analysis in SCLC patients.

Investigational Drug	Phase	Number of Patients	Blood Sample Collection	CTC Detection Method	Optimal Cut-Off	Ref
Pazopanib	Phase II	56	Baseline, after the 1st cycle and at progression	CellSearch	5 CTCs	[48]
LY2510924 plus CE	Phase II	78	Baseline, cycle 1 (day 7), cycle 2 (day 1), and at 30-day follow-up after the last dose	CellSearch	6 CTCs/7.5 mL blood	[49]
Vismodegib or cixutumumab plus CE	Phase II	120	Baseline	CellSearch	100 CTCs/7.5 mL blood	[50]
Sonidegib plus CE	Phase I	14	Baseline, after cycles 1,2,4,6, every 3 cycles during maintenance therapy and at disease progression	CellSearch	No cut-off	[51]

Abbreviation: carboplatin-etoposide (CE).
